# Protocol for the Quick Clinical study: a randomised controlled trial to assess the impact of an online evidence retrieval system on decision-making in general practice

**DOI:** 10.1186/1472-6947-6-33

**Published:** 2006-08-24

**Authors:** Enrico Coiera, Farah Magrabi, Johanna I Westbrook, Michael R Kidd, Richard O Day

**Affiliations:** 1 Centre for Health Informatics, University of New South Wales, Sydney, 2052, Australia; 2 Discipline of General Practice, University of Sydney, 37A Booth Street, Balmain Sydney NSW 2041, Australia; 3 Physiology & Pharmacology, School of Medical Sciences, Faculty of Medicine, University New South Wales, Sydney 2052, Australia; 4 St Vincent's Hospital, Victoria St., Darlinghurst, NSW 2010, Australia

## Abstract

**Background:**

Online information retrieval systems have the potential to improve patient care but there are few comparative studies of the impact of online evidence on clinicians' decision-making behaviour in routine clinical work.

**Methods/design:**

A randomized controlled parallel design is employed to assess the effectiveness of an online evidence retrieval system, Quick Clinical (QC) in improving clinical decision-making processes in general practice. Eligible clinicians are randomised either to receive access or not to receive access to QC in their consulting rooms for 12 months. Participants complete pre- and post trial surveys.

Two-hundred general practitioners are recruited. Participants must be registered to practice in Australia, have a computer with Internet access in their consulting room and use electronic prescribing. Clinicians planning to retire or move to another practice within 12 months or participating in any other clinical trial involving electronic extraction of prescriptions data are excluded from the study.

The primary end-points for the study is clinician acceptance and use of QC and the resulting change in decision-making behaviour. The study will examine prescribing patterns related to frequently prescribed medications where there has been a recent significant shift in recommendations regarding their use based upon new evidence. Secondary outcome measures include self-reported changes in diagnosis, patient education, prescriptions written, investigations and referrals.

**Discussion:**

A trial under experimental conditions is an effective way of examining the impact of using QC in routine general practice consultations.

## Background

Several studies have shown that clinicians have many unanswered questions during clinical encounters which may impact on the quality and outcomes of decisions made. Depending on measurement techniques used to estimate the frequency of questions and the clinical setting, clinicians have anywhere between 1 and 6 questions per patient encounter and answers are pursed for only about 30% to 55% of questions [1-4]. Improving access to clinical evidence through the implementation of evidence retrieval systems is one way of supporting information seeking in routine care [5]. Easy access to up-to-date evidence at the point of care may be more effective than other interventions aimed at improving clinicians adoption of evidence-based practice such as CME, distribution of clinical guidelines, clinical detailing, etc.

The effect of implementing information retrieval technology in routine clinical settings and its impact on clinicians' decision-making has not been extensively investigated. A 2005 review found that on average one in three searches for information were associated with a positive impact on decision-making, however in 73% (19 of 26) of the studies examined in this review, the impact was self-reported by clinicians using the system [6]. Assessments of evidence retrieval systems have relied principally on post study surveys. Only a small number have directly measured use in clinical settings and in most cases patterns of searching and clinical usefulness of information retrieved could not be identified.

There are few comparative studies of online evidence retrieval technology in routine clinical settings. A Finnish study found no difference in compliance with recommendations between computerised and paper-based clinical guidelines among newly qualified physicians [7]. In another study involving fourth year medical students, participants reported that access to information on a handheld computer encouraged use of information, improved learning and increased confidence in decision-making [8]. However, the effect of information retrieval technology on clinicians decision-making process and impact upon patient outcomes has not been assessed.

We are conducting a randomised controlled trial (RCT) to investigate the effectiveness of an information retrieval system called Quick Clinical (QC) in general practice [9]. The feasibility of the QC system was demonstrated in a previous clinical trial, detailed results of this study are reported in a separate paper [10]. Overall, 193 clinicians used the online evidence system to conduct on average 8.7 searches per month. The majority of these searches were conducted from consulting rooms (81%) during office hours. The most frequent searches conducted related to questions about diagnosis (40%) and treatment (35%). Search subjects included a broad spectrum of diseases, including common conditions such as asthma, diabetes and hypertension. In this paper we describe the design and proposed analysis of an RCT to evaluate the effectiveness of using QC in routine general practice consultations.

## Methods

### Study aims

The aim of this trial is to assess the effectiveness of an online evidence retrieval system (QC) in improving clinical decision-making processes in general practice.

The specific hypotheses to be tested in this study are that:

1. Online evidence systems are clinically acceptable and will be used by clinicians in 'real world' general practice settings.

2. Online evidence systems are effective in changing clinical decision-making behaviour and result in measurable improvements in evidence-based prescribing decisions.

### Study design and setting

A randomized controlled parallel design is used to evaluate the effectiveness of QC.

#### Intervention

**Figure 1:**
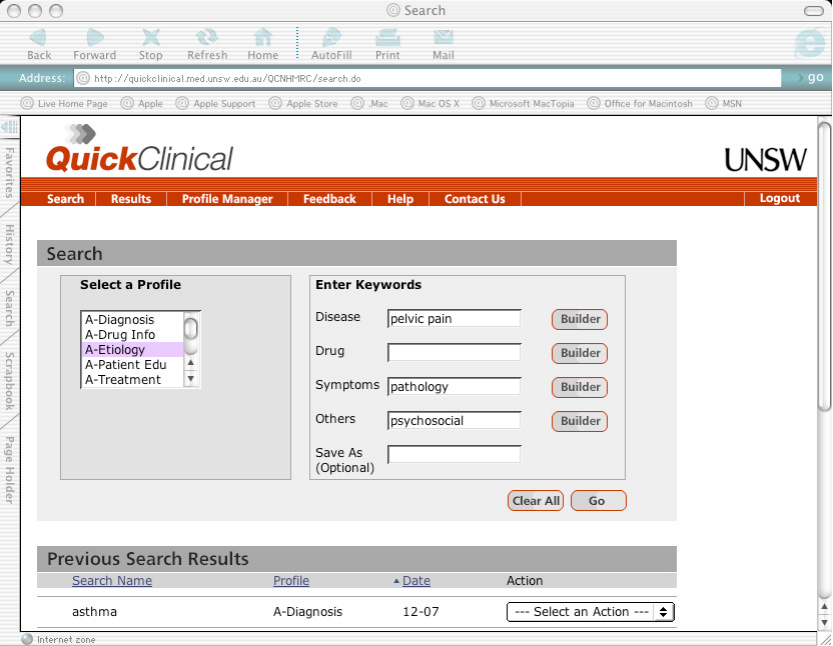
Quick Clinical user interface.

Participants are randomised to receive access to QC in their practice for 12 months. The system is available from a standard web browser e.g. Microsoft Internet Explorer, Firefox etc. (Figure 1). Each user has a personal username and password and completes an online tutorial about how to use the system. QC is based on the generic use of search filters specifically designed around the information needs of general practitioners (GP). Five types of search filters or "profiles" are available (disease aetiology, diagnosis, treatment, prescribing and patient education). Up to four keywords can be used to describe a clinical question. Clinicians search for information firstly by selecting a search filter to match their question type (e.g. diagnosis, treatment etc.) and then entering keywords to describe the query. For example, a clinician who encounters a 32-year old woman with a fourth presentation of pelvic pain in the last 6 months with ultrasound and swabs for infection all negative, may have a question regarding the social, psychological as well as biological causes of pelvic pain. The clinician could select the 'etiology' profile and enter 'pelvic pain', 'pathology' and 'psychosocial' as keywords (Figure 1). The search filters retrieve evidence from resources including Therapeutic Guidelines (a summarised evidence resource developed by Australian experts); the Merck Manual; Health Insite (a government funded health database); PubMed; MIMS (a pharmaceutical database); and a collection of relevant Australian guidelines identified by a panel of clinical experts. Users can also search each of these resources individually.

**Figure 2:**
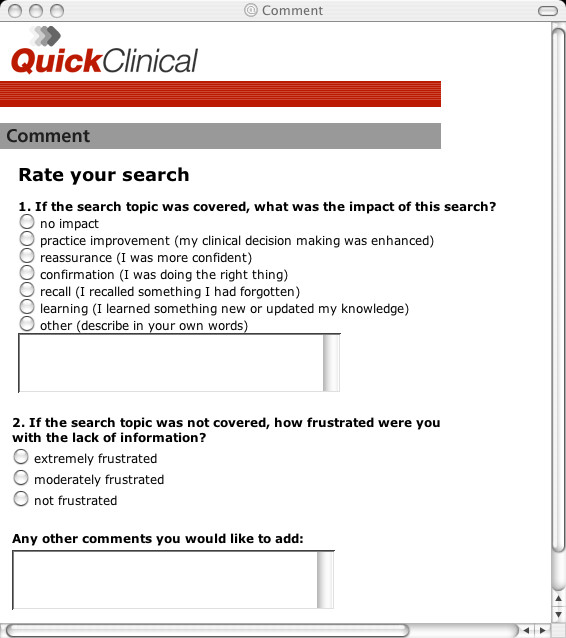
Screenshot of online feedback facility (questions after [16]).

#### Outcomes

The primary outcome measures are clinician acceptance and use of QC and the resulting change in decision-making behaviour. The data from our pilot study of QC involving 227 GPs showed that online evidence was sought in relation to both frequent and rare clinical conditions across a broad range of clinical areas [10]. This suggests that we are likely to see access to evidence having a broad impact across common and rare clinical decisions. The RCT will therefore examine prescribing patterns related to frequently prescribed medications where there has been a recent significant shift in recommendations regarding their use based upon new evidence. Examining the effect on management decisions in clinical areas where there is shifting evidence enables a hypothesis driven approach to determine the impact of online evidence on clinician behaviour.

The RCT will therefore focus on the changes in clinicians' prescribing patterns in Australian National Health priority areas identified by a panel of experts including chief investigators, associate investigators and general practitioners (Table 1). Non-pharmacological clinical management in these priority areas will also be examined e.g. referral for podiatry (diabetes management), spirometry (asthma), HbA1c testing (diabetes), cognitive behavioural therapy (CBT) for depression, dieticians for lipid disorder. In addition to changes in priority areas, prescribing patterns in response to new evidence of the effectiveness of new or existing treatments identified through reviews of literature and expert input will also be assessed. Secondary outcome measures to determine impact on decision-making behaviour include self-reported changes in diagnosis, patient education, prescriptions, investigations and referrals.

**Table 1:** Clinical priority areas used to measure primary outcomes.

***Clinical area***	***Expected changes in intervention group***
Asthma	↓ short acting beta-agonists ↑ long-acting beta-agonists ↑ inhaled corticosteroids (decreased dose of steroids over time i.e. back titration or combinations of LABA & corticosteroids)
Depression	↑ SSRIs, ↑ SNRIs ↓ TCAs;
Hypertension	↑ diuretics ↓ other antihypertensives
Upper respiratory tract infection	↓ antibiotics and ↑ appropriate antibiotics
Immunisation/vaccination	Higher rate of immunization and closer compliance with schedule.
Lipid disorders	↑ Statins,
Type 2 Diabetes	↑ glitazones ↑ metformin ↑ insulin ↓ sulfonylureas
Non-inflamatory musculoskeletal including osteoarthritis	↑ paracetamol ↓ NSAIDs ↓ Cox-2 inhibitors

#### Ethical considerations

Ethical approval for the study was obtained from the University of New South Wales's Human Research Ethics Committee and subsequently ratified by Sydney University and the Royal Australian College of General Practitioners ethics committees.

The study is also recognised by the Royal Australian College of General Practitioners (RACGP) for its continuing medical education (CME) program. Education points are not directly linked to participants' use of QC, but to trial completion.

### Identification of eligible general practitioners

**Figure 3:**
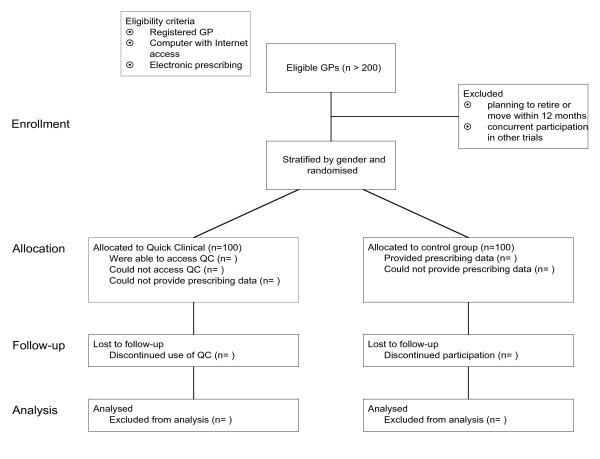
Overview of trial process.

GPs who had a computer with Internet access in their consulting rooms and used electronic prescribing are recruited via a call for volunteers advertised in journals, newsletters and a clinician list-server. An overview of the trial process is shown in Figure 3. Volunteers are asked to register via a web page and to complete a short survey to determine their eligibility to participate in the study. The online registration gathers baseline data about the demographics of the sample including practice characteristics and computer use [see Additional file 1]. Eligibility and exclusion criteria are listed in Table 2. To participate in the study clinicians must be registered to practice in Australia; have a computer with Internet connectivity; and prescribe electronically. GPs who are planning to retire or move to another practice within 12 months or participating in other concurrent trials involving electronic extraction of prescription data are excluded from the study.

**Table 2:** Study inclusion and exclusion criteria.

***Inclusion criteria***
• general practitioners registered to practice in Australia
• use a computer with Internet access in consulting rooms
• use electronic prescribing
***Exclusion criteria***
• concurrent participation in other clinical trials involving electronic extraction of prescription data
• planning to retire or move within the next 12 months

Eligible GPs are randomly allocated to either the control or intervention group, each clinician is given a unique study identification code and the web address of QC is sent to eligible participants one week prior to commencement of the trial. Participants visiting the QC web site are given detailed information about the trial along with the participant information statement and the consent form. A revocation of consent form is also available online. Clinicians willing to participate complete the online consent form and a pre-trial survey which assesses information seeking habits and attitudes to information seeking during consultations [see Additional file 2]. A psychometric instrument used to classify clinicians' response styles to new information as seekers, receptives, traditionalists, or pragmatists is also administered as part of the pre-trial survey [see Additional file 3] [11].

### Data collection

The timing and content of study assessments are summarised in Table 5. Frequency and purpose of system use of QC are determined from automatically generated computer logs used to record details of each search including the search filter chosen, keywords entered, data sources accessed, user IP (Internet Protocol) address, date, time and duration of searches [see Additional file 4]. Users are asked to identify if the IP address is their home or consulting room. An online feedback facility within the QC user interface is used to record clinicians comments in context of specific searches that generate the feedback (Figure 2).

Participants in both the control and intervention group provide prescribing data in electronic format [see Additional file 5]. The data extraction process excludes all potentially identifying information such as patient name, address, gender, postcode, telephone number, Medicare number, progress notes, images (e.g. X-rays) and any data fields that could identify a patient, such as free text fields. Data for the study period (i.e. 12 months retrospective data plus 12 months of the intervention period) is electronically extracted from prescribing software using secure means without any intrusion on clinicians' work.

The prescription data extraction software and installation instructions are sent by email and also are made available online. The first data extraction (i.e. retrospective prescriptions data from the previous 12 months) is performed after installation of the extraction software at the start of the study. The second extraction is scheduled at 6 months and the final at 12 months. An uninstall program will remove all extraction software, created files and changes to profiles in clinicians' prescribing software. At the end of 12 months all participants complete a post-trial survey [see Additional file 6]. Participants attitudes to information seeking and responses to the psychometric instrument used in the pre-trial survey are repeated post-trial.

#### Randomisation, blinding and allocation concealment

Eligible GPs are stratified by gender and randomised to the intervention or control group using a computer generated random number sequence in randomly assigned blocks (max. block size 8) and online evidence allocation ratio of 1:1. For each level of strata, a random sequence of study group assignments is generated using a computerized random-number generator [12]. Each level of strata are numbered consecutively. Each doctor receives the next consecutive number in his or her stratum.

Since QC is a behavioural intervention it is not possible to blind participants to the intervention. However investigators are blinded to group assignment. The randomisation sequence is generated and clinicians are randomly assigned to two groups by a researcher from the Evaluation team of the Centre for Health Informatics. This individual is not directly involved in the study nor its analysis.

The group assignment is revealed to clinicians online only after they consent to participate in the study following completion of the pre-trial survey. To minimise contamination of control participants who might work closely with practitioners who are part of the intervention group, only one clinician is eligible to participate in the trial from each practice. In cases where there are multiple registrations from a single practice, only the first registrant is eligible to participate. Participants in the intervention group are asked not to share their QC access details with their colleagues.

#### Communication with participants

Email will be the primary channel to communicate with participants for information and reminders about:

1. start of the trial (followed up with 2 fortnightly reminders)

2. instructions for installing and using prescribing data extraction tool

3. post-trial survey

4. education points notification and summary of main results

#### Subject withdrawal

Participants who formally withdraw from the study are contacted by telephone to determine reason for withdrawal. Similarly participants who do not use QC nor provide prescribing data are contacted to determine reasons for not completing the study.

### Sample size considerations

Sampling unit is the GP and the unit of analysis is also the GP. We provide the following example to demonstrate that the RCT sample size will be more than sufficient for detecting statistically significant prescription changes in medication areas where there is a shift in evidence. The example used to verify the design is measuring a 10% increase in use of preventer medications (glucocorticoid inhaled medications) in Asthma. Currently on average, 53% of GP encounters for asthma lead to the prescription of a preventer medication [13]. We expect that access to QC will promote the use of preventer medication by GPs who use the intervention. We expect that in the intervention group – at least 58.4% of GP encounters for asthma lead to the prescription of a preventer medication.

As the prescriptions belonging to the same GP will tend to be correlated, the sample size calculations estimated the average number of condition-specific prescriptions per GP and adjustments were made for intracluster correlation factor estimated to be 0.02 [14].

The number of GPs required in each arm to detect a 10% change in average prescription rate per 100 condition-specific encounters per GP are listed in Table 4[15]. Given that data collection in the trial will run for a minimum of 12 months 100 GPs in each arm will be sufficient to detect a 10% change in prescriptions (p = 0.05, power = 0.80).

### Statistical analyses

All registered participants will have completed the pre-trial survey. The subset of the intervention group analysed for primary outcome will have completed the post-trial survey and provided prescribing data. Participants in intervention group who encounter technical difficulties in using QC will be excluded from this analysis (i.e. those who had the opportunity to use QC but did not do so will be included). All of the control group except those who did not provide prescribing data will be included in the primary analysis. As summarised in Table 5 the intervention and control groups will be compared for baseline differences in prescription patterns, case-mix and profile of participants. Using an intention to treat analysis adjusted data will be analysed to determine differences in prescribing patterns and non-pharmacological management in identified priority areas, and areas in which there have been recent changes in response to new evidence using a one-tailed binomial test for proportions. Self-reported changes in diagnosis, patient education, prescriptions, investigations, referrals will also be examined. Utilisation and outcome measures will be analysed relative to QC usage and patient load (e.g. by number of patients seen).

### Time plan for the RCT of Quick Clinical

Recruitment commenced in October 2004, 203 GPs volunteered to participate in the study and the trial formally commenced in May 2005. The trial under experimental conditions will evaluate the effectiveness of using QC in routine general practice consultations.

## Competing interests

QC was developed by researchers at the Centre for Health Informatics at UNSW, and the university and some of the authors could benefit from commercial exploitation of QC or its technologies.

## Authors' contributions

Enrico Coiera, Johanna Westbrook, Michael Kidd and Richard Day were responsible for identifying the research question; development of the protocol and study design; and contributing to drafting of the study protocol. Farah Magrabi, as project manager, has contributed to the development of the protocol and study design. Farah Magrabi and Enrico Coiera were responsible for drafting this paper. All authors provided comments on the drafts and have read and approved the final manuscript.

**Table 3:** Timing and content of study assessments.

***Identifying eligible clinicians***
Trial registration: self completed online

***Baseline data***
Pre-trial survey: self completed online
Prescription data from previous 12 months: electronic extraction

***Clinician follow-up procedures***
Primary outcome measures: Computer log and electronic prescription data at 6 and12 months
• Physician acceptability focusing on ease of use and usefulness (post-trial survey) and patterns of QC use (computer logs)
• Prescribing patterns in clinical priority areas identified at the start of the study
• Prescribing patterns in response to new evidence of the effectiveness of new or existing treatments
• Patterns of non-pharmacological clinical management
***Secondary outcome measures: self-reported in online post-trial survey at 12 months***
• Referral patterns
• Management decisions
• Number, timing and types of investigations

**Table 4:** Sample size estimation (statistical power: 80% power, at the 5% significance level).

***Condition***	***Annual rate of medication use per 100 problem-specific encounter***	***Sample size (no. of GPs required in each arm)***
Asthma- use of preventers	53.2	54
Asthma- SA Bronchodilators	53.1	54
Depression- SSRI	41.7	85

**Table 5:** Summary of analyses for the RCT of Quick Clinical.

***Analyses to determine impact of Quick Clinical on decision-making***
1. Baseline comparisons between control and intervention groups (unadjusted data)
a) proportion of prescriptions by ATC* categories and in priority areas (using 12 months retrospective data)
b) case-mix (age-gender distribution of patients)
c) participants' profile (gender, age, place of graduation, geographic distribution, computer skills)
2. Interim analyses at 6 months
a) analyses of baseline prescribing data. Reports of interim analyses affecting the ongoing conduct of the trial and interpretation of final results
3. Using an intention to treat analysis (i.e. irrespective of patterns of QC use in the intervention group) we will include all participants in the primary analyses using adjusted data to determine:
a) broad differences in proportion of prescriptions by ATC classification
b) specific differences in proportion of prescriptions by priority area (e.g. Antibiotics prescribed for URTI)
c) differences in prescription patterns in response to new evidence of the effectiveness of new or existing treatments
d) differences in non-pharmacological treatments
e) analysis of prescription changes relative to search categories

*Anatomical Therapeutic Chemical classification system

## Pre-publication history

The pre-publication history for this paper can be accessed here:



## Supplementary Material

Additional file 1 Online registration. Summary of items in online registration. Click here for file

Additional file 2 Title: Pre-trial survey. Summary of items in online pre-trial survey. Click here for file

Additional file 3 Physician response styles. Summary of items in online pre-trial psychometric instrument used to classify physician response styles to new information as seekers, receptives, traditionalists, or pragmatists. Click here for file

Additional file 4 Title: Quick Clinical computer log. Data collected via computer logs to determine patterns of Quick Clinical use. Click here for file

Additional file 5 Title: Prescribing data. Data electronically extracted from clinicians' prescribing software. Click here for file

Additional file 6 Title: Post-trial survey. Summary of items in online post-trial survey. Click here for file
