# Supplementary material for: Protocol for the Quick Clinical study: a randomised controlled trial to assess the impact of an online evidence retrieval system on decision-making in general practice
Source: BMC Med Inform Decis Mak. 2006 Aug 24;6:33. doi: 10.1186/1472-6947-6-33 (PMC1564384; doi:10.1186/1472-6947-6-33)
Supplement: Additional file 1 — Online registration. Summary of items in online registration. [file 1472-6947-6-33-S1.doc]

Summary of items in online registration.

| ***Demographics*** |
| --- |
| 1. Age and gender |
| 1. Indicate your experience in general practice (5 options e.g. 2-5 years) |
| 1. Country of graduation for primary medical degree (7 options e.g. Australia) |
| 1. Are you a current General Practice Registrar? (yes/no) |
| 1. College fellowships held (3 options e.g. FRACGP, FACRRM) |
| 1. Accreditation status of practice (3 options accredited, registered for accreditation, none) |
| 1. Are you a member of the GPRN (General Practice Research Network)? (yes/no) |
| 1. Did you participate in the 2002 trial of Quick Clinical? (yes/no) |
| 1. Are you planning to retire or move to another practice in the next 12 months? (yes/no) |
| ***Practice characteristics*** |
| 1. Practice type (3 options solo, group, medical centre) |
| 1. Average general practice hours (direct patient care hours) work per week |
| 1. Average number of patients seen every hour |
| 1. Do you work in more than one practice? (yes/no) |
| ***Computer use, skills and information seeking*** |
| 1. Do you have a computer on your desk where you see patients? (yes/no) |
| 1. Ratings of computer skills (5 options, excellent, very good, good, fair, poor). |
| 1. Do you use a computer during consultations? (yes/no) |
| 1. For what purpose do you use a computer during consultations? (7 options prescribing, medical records, other administrative (e.g. appointments), patient education, email, looking up evidence (e.g. journals/guidelines), Internet) |
| 1. Speed of your Internet connection (4 options not sure, 28k/33k modem, 56k modem/ ISDN, broadband (ADSL / Satellite/ Cable) |
| 1. Do you search for clinical information during consultations? (yes/no) |
| 1. Do you use a computer program for prescribing? (yes/no, indicate version) |
