# Supplementary material for: Protocol for the Quick Clinical study: a randomised controlled trial to assess the impact of an online evidence retrieval system on decision-making in general practice
Source: BMC Med Inform Decis Mak. 2006 Aug 24;6:33. doi: 10.1186/1472-6947-6-33 (PMC1564384; doi:10.1186/1472-6947-6-33)
Supplement: Additional file 2 — Title: Pre-trial survey. Summary of items in online pre-trial survey. [file 1472-6947-6-33-S2.doc]

Summary of items in online pre-trial survey.

| ***Information seeking habits*** |
| --- |
| 1. On average how often do you have a clinical question for which you feel it is necessary to seek information?  ( 5 options always to never) |
| 2. Of these clinical questions I actively seek answers for (5 options all questions to few questions) |
| 3. Three information sources used most frequently to answer clinical questions? (12 items, human, print and electronic) |
| 4. When do you usually seek information to answer your clinical questions? (3 options during consultations, between consultations, outside clinical hours) |
| 5. How often do you find answers to your clinical questions? (5 options all to none of the time) |
| 6. Attitudinal items (5 options, strongly agree, agree, undecided, disagree, strongly disagree)   - During a consultation I think it is appropriate to search for information. - My patients expect me to have the answers to all their questions. - During a consultation patients expect me to look for information if needed. - During a consultation I do not have enough time to search for information. - Using a computer during consultations usually interferes with the relationship with my patient. - Using a computer during consultations usually enhances my relationship with patients - My patients are increasingly asking for written information. - If I had sufficient time, most of my clinical questions could be answered by searching the literature. - My patients are better informed by information they have found on the Internet. - I feel my patients are more comfortable using the computer to find information than I am. - My patients are often misinformed by information they find on the Internet. |
