# Supplementary material for: Protocol for the Quick Clinical study: a randomised controlled trial to assess the impact of an online evidence retrieval system on decision-making in general practice
Source: BMC Med Inform Decis Mak. 2006 Aug 24;6:33. doi: 10.1186/1472-6947-6-33 (PMC1564384; doi:10.1186/1472-6947-6-33)
Supplement: Additional file 3 — Physician response styles. Summary of items in online pre-trial psychometric instrument used to classify physician response styles to new information as seekers, receptives, traditionalists, or pragmatists. [file 1472-6947-6-33-S3.doc]

Summary of items in online pre-trial psychometric instrument used to classify physician response styles to new information as seekers, receptives, traditionalists, or pragmatists [11]

| ***Response styles to new information*** |
| --- |
| 1. Clinical experience is more important than randomised controlled trials. |
| 1. I am comfortable practicing in ways different than other doctors. |
| 1. Evidence-based medicine makes a lot of sense to me. |
| 1. I don’t have the time to read up on every practice decision. |
| 1. It is best to change the way I treat a certain problem when my local colleagues are making the same changes. |
| 1. I follow practice guidelines if they are not too much (of a) hassle. |
| 1. The opinions of respected authorities should guide clinical practice. |
| 1. I am too busy taking care of patients to keep up with the recent literature. |
| 1. Clinical experience is the most reliable way to know what really works. |
| 1. I am uncomfortable doing things differently from the way I was trained. |
| 1. I am often critical of accepted practices. |
| 1. Patient care should be based where possible on randomised controlled trials, rather than the opinions of respected authorities. |
| 1. My colleagues consider me to be someone who marches to my own drum. |
| 1. I follow practice guidelines as long as they don’t interfere too much with the flow of patients. |
| 1. It is not prudent to practice out of step with other physicians in my area. |
| 1. The best practice guidelines are based on the results of randomised controlled trials. |
| 1. Evidence-based medicine is not very practical in real patient care. |
| (5 options strongly agree, agree, neutral, disagree, strongly disagree) |
