# Supplementary material for: Protocol for the Quick Clinical study: a randomised controlled trial to assess the impact of an online evidence retrieval system on decision-making in general practice
Source: BMC Med Inform Decis Mak. 2006 Aug 24;6:33. doi: 10.1186/1472-6947-6-33 (PMC1564384; doi:10.1186/1472-6947-6-33)
Supplement: Additional file 4 — Title: Quick Clinical computer log. Data collected via computer logs to determine patterns of Quick Clinical use. [file 1472-6947-6-33-S4.doc]

Data electronically extracted from clinicians' prescribing software.

| ***Prescription data fields*** |
| --- |
| Patient demographics (e.g. gender, age with day of birth recoded to the 15th of each month for all patients) |
| Prescriptions (e.g. drug, dosing, regime) |
| Conditions treated |
| History of diagnosis |
| Date of death |
| Requests for imaging (e.g. scans, X-rays) and pathology (e.g. lipid levels, biochemistry) |
| Clinical measurements (e.g. blood pressure, height, weight), risk factors (e.g. alcohol, smoking, drug allergies) |
| Patient management (e.g. procedures, immunisation record, INR) |
