# Supplementary material for: Protocol for the Quick Clinical study: a randomised controlled trial to assess the impact of an online evidence retrieval system on decision-making in general practice
Source: BMC Med Inform Decis Mak. 2006 Aug 24;6:33. doi: 10.1186/1472-6947-6-33 (PMC1564384; doi:10.1186/1472-6947-6-33)
Supplement: Additional file 6 — Title: Post-trial survey. Summary of items in online post-trial survey. [file 1472-6947-6-33-S6.doc]

Summary of items in online post-trial survey.

| ***Use and impact of QC*** |
| --- |
| 1. Did you use QC? (yes/no) |
| 1. Frequency of QC use in an average week. (8 options zero to every day) |
| 1. When did you mainly use QC? (3 options during consultations, between consultations outside practice hours) |
| 1. Impact of QC on length, quality of consultations, care given and focus on patient (3 options increased, decreased, did not change) |
| 1. Did you use QC at home? (yes/no) |
| 1. How often did you find the information you wanted (5 options all to none of the time) |
| 1. Ratings of skill in using QC to find information (5 options, excellent, very good, good, fair, poor) |
| 1. Reasons for using QC (12 options e.g. to confirm a clinical decision) |
| 1. How often did the information you found result in a change in patient diagnosis and management? (5 options all to none of the time) |
| 1. Do you believe QC has the potential to improve patient care? (yes/ no don’t know) |
| 1. Do you have direct experience of QC resulting in improved patient care? (yes/no) |
| 1. What impact has QC access had on your patient care decisions? Please indicate which types of decisions were affected (5 options, diagnosis, patient education, prescriptions, investigations, referrals) |
| 1. Ratings of Quick Clinical attributes [comprehensiveness of information, speed, reliability, relevance & accuracy of evidence, ease of use, presentation of results] (5 options, excellent, very good, good, fair, poor) |
| 1. Indicate your level of agreement with the following statements: |
| (5 options, strongly agree, agree, undecided, disagree, strongly disagree) |
| QC has enabled me to practice more evidence-based medical care |
| I think that QC is appropriate for use during consultations |
| QC was a valuable resource |
| I would not recommend QC to colleagues |
| I want to continue using QC |
| I would have used QC less if it didn’t give me CME points |
| ***Other educational activities*** |
| 1. Current accreditation status of practice (3 options accredited, registered for accreditation, none) |
| 1. Indicate use of other online clinical information resources in the last 12 months (8 other online resources e.g. InfoPOEMs) |
| 1. In the last 12 months did you participate in any educational activities such as clinical audits, case studies or self-audits? (yes/no) |
| 1. What factors have changed your prescribing decisions in the last 12 months? |
